# Understanding the performance of HIV-1 viral vector vaccines: adenovirus and poxvirus case studies

**DOI:** 10.3389/fimmu.2025.1720342

**Published:** 2026-01-14

**Authors:** Mahdiyeh M. Manafi, Touraj Farzani, Nallely Espinoza, Al Ozonoff, Pardis C. Sabeti

**Affiliations:** 1 Department of Exercise and Health Sciences, Manning College of Nursing and Health Sciences, University of Massachusetts Boston, Boston, MA, United States; 2 Broad Institute of Massachusetts Institute of Technology (MIT) and Harvard, Cambridge, MA, United States; 3 Department of Organismic and Evolutionary Biology, Harvard University, Cambridge, MA, United States; 4 Zoonotic and Emerging Disease Research Unit, National Bio and Agro-Defense Facility, Agricultural Research Service, United States Department of Agriculture, Manhattan, KS, United States; 5 Harvard Medical School, Boston, MA, United States; 6 Howard Hughes Medical Institute, Chevy Chase, MD, United States; 7 Department of Immunology and Infectious Diseases, Harvard T.H. Chan School of Public Health, Harvard University, Boston, MA, United States

**Keywords:** adenoviral, antibody response, clinical trial, HIV-1, poxviral

## Abstract

Despite decades of research, HIV-1 continues to infect millions annually, underscoring the urgent need for a safe and effective vaccine to curb the ongoing global pandemic. Among the many strategies explored, viral vectors have been the most intensively studied, with adenoviral and poxviral platforms serving as the leading approaches. These vectors have advanced through extensive preclinical evaluation and multiple large-scale clinical trials, demonstrating safety and the ability to induce cellular and humoral responses. Yet, they have also revealed key challenges, including pre-existing vector immunity, limited durability of responses, and in some cases, increased susceptibility to infection. Importantly, these trials clarified the limitations of Env-focused immunity, highlighted the value of heterologous prime–boost regimens, and reinforced the dual need for broadly neutralizing antibodies and functional T cell responses. While vector-based COVID vaccines achieved protective efficacy, lessons learned from adenoviral and poxviral efforts continue to shape the field, directly informing the design of next-generation platforms such as mRNA and engineered viral vectors.

## Introduction

Since its emergence in the early 1980s, the HIV-1 pandemic has remained one of the most pressing global health challenges. Despite decades of intensive research, there is still no curative therapy or effective vaccine against HIV-1. Antiretroviral therapy (ART) and pre-exposure prophylaxis (PrEP) have markedly improved outcomes and prevention, but they fall short of offering a definitive solution and remain inaccessible to many in low- and middle-income countries (1). Classical vaccine approaches that succeeded for other viruses have not translated to HIV-1. Inactivated whole-virus vaccines for this virus were abandoned due to safety concerns and technical limitations (2–4). Early therapeutic vaccines such as *Remune*, a gp120-depleted inactivated HIV-1 preparation, initially showed promise but failed to demonstrate clinical benefit in phase III trials (5). Live attenuated vaccine strategies, inspired by naturally occurring nef-deleted strains, raised unacceptable risks of persistent infection and genomic integration (6). Together, these outcomes underscored that HIV’s biology poses unique challenges for traditional vaccine paradigms. The central barrier to an HIV-1 vaccine is the virus’s extraordinary genetic diversity. HIV-1 evolves rapidly through high rates of mutation and recombination, producing a swarm of closely related variants (quasispecies) that evade immune recognition. Reverse transcriptase introduces mutations at ~1 per genome per cycle, while host mutagenic factors such as APOBEC3G and frequent recombination further accelerate divergence. This evolutionary agility makes it difficult to train the immune system to recognize and neutralize the virus consistently (7). Together, the failures of inactivated, therapeutic, and live attenuated strategies underscored that HIV’s biology demanded alternative solutions, prompting researchers to explore recombinant viral vectors as vaccine platforms (8). Recombinant DNA technology enabled engineering of viral bacKbpones to deliver HIV antigens safely and efficiently. Such vectors mimic natural infection without causing disease, eliciting strong cellular and humoral responses. Among the many viral vectors tested, adenoviruses and poxviruses became the most extensively studied, advancing from preclinical models into large-scale clinical trials (9). In this review, we summarize lessons learned from adenoviral- and poxviral-based HIV-1 vaccine efforts (**Table 1**). We highlight their immunogenicity, advantages, and limitations, and discuss how these experiences have shaped next-generation vaccine design aimed at achieving durable protection.

**Table 1:** Comparative features of adenoviral and poxviral vector-based HIV-1 vaccines.

Feature	Adenoviral vectors (Ad5, Ad26, ChAd, Hexon-chimeric)	Poxviral vectors (MVA, ALVAC, NYVAC)
Safety	Generally safe; rare concerns with Ad5 in specific populations (e.g., STEP trial). — Adenoviral vectors have acceptable safety profiles in many trials, but the STEP/Ad5 experience showed an unexpected increased HIV acquisition risk in some Ad5-seropositive men, so caution and population selection matter.	Excellent safety: non-replicating, well tolerated even with repeated dosing. — MVA/ALVAC/NYVAC have repeatedly shown good safety in humans, including repeat dosing and boosts. (Some engineered/replicating variants are being explored and require separate safety assessments.)
Antibody responses	Induce Env-binding antibodies, but generally weak neutralizing activity; durability improved with Ad26/protein boosts. — Adenoviral platforms elicit binding Abs and T-cell responses; neutralization breadth/titers are typically limited unless combined with protein boosts or optimized immunogens (Ad26 + protein strategies show improved durability).	Robust Env-binding antibody induction (often gp41-biased); neutralizing activity typically weak. — Poxviral vectors reliably induce binding antibodies and can bias certain specificities; neutralizing breadth is generally modest unless included in a heterologous prime–boost with protein or other immunogens (RV144/ALVAC + AIDSVAX gave modest protection correlated with binding Ab signals).
CD4+ T cell responses	Induce helper responses but skewed toward CD8^+^ T cell dominance. — Adenoviral vectors are potent CD8 inducers; CD4 help occur but magnitude/polarization depends on vector, antigen, and regimen.	Strong CD4^+^ T cell induction, supporting antibody production and boosting regimens. — Poxviruses (MVA/ALVAC/NYVAC) elicit CD4 help that supports humoral responses and prime–boost strategies.
CD8+ T cell responses	Potent, polyfunctional CD8^+^ T cell responses with high IFN-γ secretion; durable and cross-clade recognition (Ad26, ChAd). — Adenoviral vectors (including chimpanzee Ad) are well documented to drive strong, polyfunctional CD8 responses. Durability can be good, especially with certain serotypes/regimens.	Strong CD8^+^ T cell responses; generally lower magnitude than adenoviruses. — Poxviral vectors also elicit CD8 responses but in many studies the peak magnitude is lower than that seen with adenoviral vectors.
IFN-γ production	Strong, particularly from CD8^+^ T cells (robust ELISPOT responses). — Adenoviral vaccines produce high ELISPOT IFN-γ readouts in many trials.	Detectable, but lower magnitude IFN-γ responses compared to adenoviruses. — Poxviral ELISPOTs are commonly positive but often of lower magnitude.
Durability of responses	Durable T cell responses; antibody durability enhanced with protein boosts. — Adenoviral vectors can induce long-lived T cell memory; humoral durability is substantially improved when combined with protein boosts (or optimized regimens).	Antibody and T cell responses often decline without boosting. — Non-replicating poxviral responses wane over time and typically require boosting (protein or vector) to maintain or broaden responses.
Vector immunity	Pre-existing anti-Ad antibodies (especially Ad5) can dampen responses; newer serotypes (Ad26, ChAd) mitigate this. — Widespread Ad5 seroprevalence and anti-vector immunity are well documented; alternative serotypes and chimpanzee Ad reduce but do not eliminate the issue.	Minimal pre-existing immunity in most populations; prior smallpox vaccination may influence older individuals. — Most people lack high titers to MVA/NYVAC; but historical vaccinia exposure (older cohorts) can influence responses.
Best use	Inducing strong CD8^+^ T cell immunity; heterologous prime boost with proteins or other vectors. — Adenoviral vectors are commonly used for cellular immunity primes and in heterologous regimens with proteins to drive antibodies.	Safe priming of CD4^+^ T cells and antibody responses; effective in heterologous prime–boost regimens. — Poxviral vectors are widely used as safe priming vectors for humoral-focused regimens or as boosts in multi-component strategies.

## HIV-1 structure

Understanding HIV-1 structure is central to vaccine design. Since its identification as the cause of AIDS in 1984, extensive research has mapped its architecture and immune interactions (10). HIV-1 is a 100–150 nm retrovirus with two positive-sense RNA strands that are 5′-capped and 3′-polyadenylated, encoding nine open reading frames and 15 proteins, including structural (MA, CA, NC, p6), envelope (gp120, gp41), enzymatic (PR, RT, IN), and regulatory/accessory proteins (Vif, Vpr, Nef, Tat, Rev, Vpu) (11). Replication involves reverse transcription and integration of the proviral DNA flanked by long terminal repeats (LTRs, ~640 bp) that regulate transcription (12). HIV-1 is divided into groups M, N, O, and P; Group M drives the global pandemic and includes multiple subtypes and recombinant forms, largely diversified through the envelope glycoprotein (Env), the main target of neutralizing antibodies (13). Within hosts, HIV circulates as a quasispecies due to high mutation and recombination rates—template switching by reverse transcriptase occurs about once every 2 Kbp (14, 15). This genetic plasticity complicates immune recognition, underscoring the need to identify conserved epitopes critical for vaccine development (16).

### Immune responses against HIV-1

#### Antibody response

Antibodies are central to immune defense against HIV-1, but their timing, specificity, and function differ markedly (17). Early infection elicits Env-binding antibodies that fail to control viremia, whereas later responses in some individuals mature into broadly neutralizing antibodies (bNAbs) with exceptional potency (18). Understanding this continuum is key to explaining HIV pathogenesis and the difficulty of eliciting protective antibodies, forming the basis for vaccine design (10). The first detectable antibodies target gp120, particularly the immunogenic V3 loop, but do not reduce viral load or drive envelope evolution. Over time, some individuals develop bNAbs that neutralize diverse heterologous strains (19).

HIV antibodies can be grouped into three classes (20): i) Non-neutralizing antibodies (nNAbs): Unable to block infection even against autologous viruses, they mediate Fc-dependent effector functions such as ADCC (via NK cells) and ADCP (via monocytes, macrophages, or dendritic cells) (21). These functions are influenced by IgG subclass and Fc glycosylation; notably, afucosylated Fc glycans enhance ADCC (22). ii) Strain-specific neutralizing antibodies: These can transiently suppress replication but rapidly select for resistant variants through mutations, insertions/deletions, or glycan shifts in gp120 variable loops (23). iii) Broadly neutralizing antibodies (bNAbs): These neutralize diverse HIV-1 strains at low concentrations (IC50 <1 μg/ml) by targeting conserved Env regions such as the CD4 binding site, V1/V2 apex, V3 base, MPER, gp120–gp41 interface, silent face, and fusion peptide. Their breadth arises after years of affinity maturation and extensive somatic mutation, overcoming Env variability and glycan shielding (24–26).Because only bNAbs exhibit broad and durable activity, the majority of vaccine efforts focus on understanding their natural development (27).

#### T Cell response

T cells are central to HIV pathogenesis, yet their roles reveal paradoxes that complicate vaccine development (28). Early studies showed that despite severe CD4^+^ T cell depletion—the primary targets of HIV—potent CD8^+^ T cell responses persist in blood and mucosal tissues, even during advanced AIDS (29, 30). This dominance of CD8^+^ over CD4^+^ activity likely reflects HIV’s preferential destruction of memory CD4^+^ T cells in lymphoid-rich sites, such as the gastrointestinal tract, through direct infection and bystander apoptosis during immune activation (31). Although HIV targets activated memory CD4^+^ T cells, only a fraction become productively infected, underscoring that activation alone is insufficient for permissiveness. HIV-specific CD4^+^ T cells represent a notable exception, as repeated antigen-driven activation in virus-rich tissues renders them preferential targets for infection and depletion. By contrast, other activated memory CD4^+^ T cells may experience transient or spatially shielded activation states that limit productive infection (32).

Vaccine platforms like DNA and poxvirus-based vectors tend to induce stronger CD4^+^ than CD8^+^ responses, underscoring the challenge of replicating the balanced T cell dynamics seen in natural infection (33). CD8^+^ T cells are indispensable for HIV control throughout infection. Their antiviral effect arises from both cytolytic and non-cytolytic mechanisms. During acute infection, rapid viral escape within days of transmission supports cytolysis as the main mode of action, since CD8^+^ T cells expressing high perforin levels efficiently kill infected cells (34, 35). Non-cytolytic control also contributes: β-chemokines (CCL3, CCL4, CCL5) inhibit viral entry by blocking or downregulating CCR5. However, these mechanisms do not clear infected cells, and kinetic models suggest direct killing predominates in acute infection. In contrast, non-cytolytic pathways such as IFN-γ–mediated suppression can control other viruses like hepatitis B (36). In SIV-infected macaques, CD8^+^ depletion during chronic infection sometimes fails to change infected cell lifespan, suggesting diverse control mechanisms (37). Yet other studies link CD8^+^ depletion in HIV or SIV controllers to loss of viral control, while some find no correlation between CD8^+^ responses and clearance (38–45). As infection transitions into the chronic phase, CD8^+^ T cells diversify functionally. Beyond cytolysis, polyfunctional CD8^+^ T cells capable of secreting IL-2, IFN-γ, and TNF-α correlate with long-term control, especially in elite controllers and non-progressors (46). IL-2 production supports memory maintenance and may depend on preserved CD4^+^ help. Similar patterns are seen in HIV-2 infection, often associated with superior control (46). Overactivation of CD4^+^ T cells pose risks for vaccination (47). Excessive CD4^+^ activation without adequate CD8^+^ induction can enhance HIV susceptibility, as seen in the STEP trial, where elevated CD4^+^ activation correlated with higher infection rates (48, 49).

Preexisting HAdV-5immunity expanded activated CD4^+^ populations, increasing target cells for HIV, highlighting the need to balance CD4^+^ and CD8^+^ responses (50). Genetic evidence underscores T cell importance: specific HLA class I alleles correlate with better viral control, and escape mutations reflect viral adaptation to CD8^+^ pressure (51).

CD4^+^ T follicular helper (TFH) cells are essential for developing high-affinity antibodies and supporting memory CD8^+^ T cells. Reductions in viremia during acute infection correlate with CD8^+^ expansion and stronger antibody responses, indicating that an effective vaccine must induce both bNAbs and functional CD8^+^ T cells (52, 53). Collectively, studies of HIV-specific T cell responses highlight their crucial role in viral control and the barriers to translating this into vaccines—insights largely drawn from HIV controllers, in whom defined CD8^+^ CTL responses and protective HLA alleles are associated with low viral loads (54–56).

### Env protein structure as the main target for vaccine design

The HIV-1 Env glycoprotein is the primary target of neutralizing antibodies and the basis of most vaccine designs (57). Env is synthesized as a 160 kDa precursor (gp160) that forms trimers of noncovalently linked gp120–gp41 heterodimers. Gp120, a heavily glycosylated surface subunit, binds CD4 and coreceptors CCR5 or CXCR4, while gp41 anchors Env to the membrane and mediates fusion. Proper gp160 cleavage by host furin is essential for infectivity (58, 59). Env’s dense glycosylation and sequence variability shield it from immune recognition (60). Gp120 is nearly half glycans by mass and contains five conserved (C1–C5) and five variable (V1–V5) regions, organized into four domains: inner and outer regions, a bridging sheet, and the V1/V2 domain (61). The conserved domains mediate receptor binding and gp41 association, while variable loops—particularly V1/V2—modulate coreceptor usage, neutralization sensitivity, and immune escape (62). V1/V2 epitopes fall into three classes (V2q, V2p, V2i) recognized by distinct monoclonals; despite lower immunogenicity than V3, V1/V2-targeting antibodies have shown protective activity in SIV and HIV challenge models (63–65).

The V3 loop, about 35 amino acids long and stabilized by a disulfide bond, directs CCR5 binding after CD4 engagement (66). It elicits potent neutralizing antibodies such as 447-52D, which recognizes GPGR motif at amino acids 312–315 on the tip of V3 loop (67). Although often occluded within the trimer, conserved elements of V3 become exposed when V1/V2 or V3 stems are shortened, increasing neutralization sensitivity (68). V3 overlaps receptor-binding surfaces, so modifications can also expose the CD4 binding site; heavy glycosylation further protects it, illustrating its dual role in immunogenicity and immune evasion (69). Broadly neutralizing antibodies (bNAbs) achieve potency by targeting conserved Env regions that are functionally constrained, including the membrane-proximal external region (MPER) of gp41, glycan-dependent sites in V2/V3, and the CD4 binding site. Potent human monoclonals such as PGT121–131 recognize V3 glycans at positions 301 or 332 (26). In chronic infection, MPER- and V3-directed responses often emerge, making them key vaccine targets. However, autoreactivity and tolerance mechanisms can limit the maturation of B cells required for these antibodies (24, 70).

Compared to gp120, gp41 is smaller, less variable, and less glycosylated, making it an appealing bNAb target. Its MPER contains linear epitopes recognized by potent antibodies such as 2F5, Z13e1, 4E10, and 10E8, though vaccines rarely elicit them reliably. Limitations include weak helper T-cell epitopes and immunodominance of other Env regions (71–73).

Among conserved Env sites, the CD4 binding site (CD4bs) remains one of the most promising vaccine targets (74). This essential and conserved region is recognized by antibodies such as b12 (neutralizing ~35% of isolates) and by newer antibodies like VRC01, 3BNC117, and NIH45-46, which neutralize >90% of strains (75, 76).

It is worth mentioning that gp41 expression can directly suppress T-cell immunity. gp41 on peptide-pulsed target cells inhibits IFN-gamma and CD25 expression in antigen-specific CD8^+^ T cells, suggesting it modulates cytokine release and dampens activation, contributing to HIV-1 immunopathogenesis. This suppression originates from its cytoplasmic tail (CT), specifically the lentiviral lytic peptide 2 (LLP2) region, which inhibits T-cell proliferation, cytokine secretion, and CD69 expression by impairing Akt signaling downstream of the TCR complex (77–79).

Overall, a major obstacle in HIV-1 vaccine development is the absence of well-defined immune correlates that reliably predict protection, hindering our understanding of the immune responses required to block infection. Challenges include the virus’s extraordinary diversity, high mutation rate, and rapid establishment of latent reservoirs that evade immunity. Env remains central to HIV-1 vaccine design: despite variability and glycan shielding, it contains conserved epitopes—V3 glycans, MPER, and CD4bs—targeted by potent bNAbs (80).

Immunogen design has explored different approaches like mosaic and consensus strategies to broaden recognition. Mosaic proteins combine optimized epitopes and by using computational tools, this strategy identifies viral genes or proteins whose sequences best reflect the diversity of circulating HIV−1 strains globally, while consensus sequences represent the most common amino acids at each site (81). For group M, two consensus Env sequences—Consensus S (ConS) and Consensus M (ConM)—were developed from all or subtype-specific sequences, respectively (82). Both display favorable immunogen traits, including minimized rare residues, reduced glycan holes, and shorter variable loops, which help focus responses on conserved epitopes (83). These approaches alongside other methods like centralized and conserved T-cell vaccine design, mapping the co-evolution of broadly neutralizing antibodies (bnAbs) with HIV, uncovering B-cell lineage cooperation in bnAb development, resolving HIV Env trimer structures, and eliciting epitope-focused Env immunogens to drive bnAb responses will help us to design more effective vaccine candidates for HIV-1 (84).

### Early HIV vaccines

Early vaccines against HIV-1 began with traditional approaches, such as inactivated or attenuated pathogens, but these strategies proved largely unsuccessful. Inactivated whole-virus vaccines showed some promise in experimental models but were undermined by safety concerns, including incomplete inactivation and inconsistent production (85). For example, formaldehyde-inactivated HIV-1 induced immune responses in murine studies, yet sera from vaccinated animals caused cytotoxic effects that complicated interpretation (86). Therapeutic vaccine strategies were also pursued, designed to modulate host immunity in infected individuals rather than prevent transmission (87). The most prominent, Remune, was derived from a chemically modified, gp120-depleted form of inactivated HIV-1. While early clinical studies suggested reductions in viral load when combined with ART, the pivotal phase III trial launched in 1997 failed to show significant benefit over placebo and was discontinued (88). Live attenuated vaccines generated significant early interest for their ability to induce durable immunity. Observations of natural infection with nef-deleted HIV-1 variants suggested partial control of viral replication, but such strains still caused immunological damage, raising major safety concerns (89, 90).

The capacity of HIV-1 to integrate proviral DNA into the host genome further amplified the risks, as latent reservoirs could be established and persist indefinitely. These hazards effectively precluded live attenuated HIV-1 vaccines from further development (91). The shortcomings of early vaccine efforts underscored the extraordinary challenges posed by HIV-1 biology. Chief among them is the virus’s extreme genetic diversity, driven by its rapid mutation rate and frequent recombination (92). Within each host, HIV-1 exists as a swarm of related variants, or quasispecies, that enable immune evasion under selective pressure. The viral reverse transcriptase lacks proofreading capacity, introducing mutations at an estimated rate of 1.4 × 10^−5^ errors per base pair per replication cycle—approximately one mutation per genome per cycle (93). Additional variability is introduced by host mutagenic enzymes such as APOBEC3G, while recombination between quasispecies accelerates adaptation and facilitates the emergence of drug-resistant or immune-escape strains (94). This extraordinary evolutionary capacity complicates vaccine design by continuously generating divergent antigenic variants that escape recognition (94). Faced with these limitations, researchers began to explore recombinant viral vector platforms as an alternative strategy for eliciting protective immunity (95).

### Adenoviruses as vaccine platform for HIV-1

#### Human adenovirus biology

Human adenoviruses (HAdV) are widely used as viral vectors in vaccine research, including in HIV-1 clinical trials, because of their stability, genetic tractability, and ability to induce potent immune responses (96). They are non-enveloped, icosahedral viruses with a linear double-stranded DNA genome ranging from 26 to 45 Kbp, most commonly about 36 Kbp. The genome is organized into early (E1–E5) and late (L1–L5) transcription units (97). Early genes regulate host cell processes required for viral replication, while late genes encode structural proteins such as hexon and fiber that form the capsid and mediate virus assembly and release (98–101). Additional coding regions outside the canonical early/late classification contribute to viral replication and stability. These include protein IX (pIX), which stabilizes the virion; virus-associated RNAs I and II, which enhance protein synthesis and the U exon protein (UXP), which contributes to DNA replication and RNA transcription (102–104).

During adenovirus infection of a host cell, the earliest gene expressed is E1A, which is activated by host transcription factors and initiates the expression of other early genes, including E2, E3, and E4 (105). E1B plays complementary roles by promoting nuclear export of late mRNA, mediating degradation of host proteins, and supporting cellular transformation (106). Transition to the late phase is regulated by the major late promoter (MLP) and L4 proteins, which coordinate expression of the capsid proteins necessary for virion assembly (107). The first generation of replication-deficient adenoviral vectors was generated by deleting the entire E1 region (E1A and E1B), thereby abolishing viral replication (108, 109). In producer cell lines, E1 function can be supplied in trans, allowing efficient propagation of E1-deleted vectors. Additional deletion of the E3 region, which is non-essential for replication, further increases packaging capacity, expanding the space available for transgene insertion from roughly 4.5–4.7 Kbp with E1 deletion alone to about 8 Kbp with combined E1/E3 deletions (110, 111).

Despite their utility, first-generation vectors, particularly those based on adenovirus serotype 5 (HAdV-5), presented several limitations that shaped subsequent development. Standard E1/E3-deleted vectors retain most of the adenoviral genome, meaning that many viral proteins are still expressed in transduced cells. These antigens are presented on MHC class I and II, leading to cytotoxic T-cell recognition and clearance of transduced cells within 2–3 weeks (112).

Pre-existing humoral immunity further reduces efficacy, especially antibodies targeting the solvent-exposed hypervariable regions (HVRs) of the hexon protein and the fiber knob, which limit transgene expression. Neutralizing antibodies typically blocks viral infection, but interestingly, some can bind adenoviruses without preventing cellular entry, complicating predictions of vector performance (113).

Other host immune mechanisms also constrain adenovirus-based vaccines. The cytosolic Fc receptor TRIM21 can recognize antibody-coated virions, promote vector clearance but also diminish the quality of antigen-specific cytotoxic T lymphocyte (CTL) responses to HAdV-5 (114). In addition, repeated adenoviral immunizations generate cross-reactive, serotype-specific antibodies that, while often non-neutralizing, can reduce transgene expression through Fc-dependent mechanisms such as antibody-dependent cellular cytotoxicity, complement activation, or opsonization, as well as through Fc-independent pathways (50). T cell responses directed against conserved epitopes in the C-terminal region of hexon proteins further reduce vector effectiveness due to cross-reactivity across serotypes. Finally, innate immune pathways also play a role: depletion studies in animal models suggest that natural killer (NK) cells limit adenoviral transgene expression, underscoring the need to account for innate as well as adaptive immunity in adenovirus-based vaccine design (115).

#### Human adenovirus 5-based HIV-1 vaccine candidates

Recombinant human adenovirus type 5 (HAdV-5) vectors were long considered promising HIV-1 vaccine platforms because they are highly immunogenic, capable of expressing large amounts of antigen, and relatively straightforward to manufacture. Preclinical studies confirmed these strengths, and early phase I/II clinical trials (**Table 2**) showed acceptable safety and the induction of cellular immune responses, setting the stage for large-scale efficacy testing (116).

**Table 2:** Adenoviral-based HIV-1 vaccines.

Trial	Clinical trial ID	Vaccine platform	Phase	Start-end	Cellular response	Humural response	Ref
STEP (v520-023)	NTC00095576	MRK AD5 HIV-1 Gag/Pol/Nef	Phase II (IIb)	2004-2009	IFN-gamma ELISPOT	Increase HIV infection risk in MSM and circumcised men	(117)
Phambili (HVTN503)	NCT000413725	MRK Ad5HIV-1 Gag/Pol/Net	Phase II (IIb)	2007-2012	IFN-gamma ELISPOT to clade B and C antigen	No efficacy	(118)
VRC 012	NTC000479999	VRC-HIVADV027-00-VP (rAd35-EnvA) and VRC-HIVADV038-00-VP (rAd5-EnvA)	Phase I	2007-2014	Env-specific ELISPOT IFN-gamma, More CD8+ T cells in Ad5 prime and rAd35 boost.	higher Binding antibody in single Ad5, More antibody in heterologous regime	(135)
IAVI B003/IPCAVD-004	NTC01215149	Ad26-ENVA.01, Ad35-env	Phase I	2010-2016	Modest T cells response (IFN-gamma ELISPOT and ICS)	Env-binding antibody responses	(137)
HVTN083	NTC01095224	rAd35EnvA, rAd5 EnvA, rAd5EnvB	Phase I	2010-2016	T cell response: IFN-gamma-ELISPOT and ICS (IFN-gamma, IL-2), CD8+>CD4+	Binding antibody responses, Low IgA binding to ConS gp140, very weak Nab	(139)
IPCAVD 001	NTC00618605	Ad26 Env.01 Expressing clade A Env gene (modified gp140)	Phase I	2008-2015	IFN-gamma ELISPOT and ICS: i) Central memory [CD27+, CD45RO+, ii) Effector memory [CD27-, CD45RO+ for both CD4+ and CD8+ T cells.	NA	(142)
Imboko/HVTN705/HPX2008	NTC03060629	Ad26.Mos4.HIV expressing mosaic env, gag and Pol + Clade C gp140	Phase IIb	2017-2008	Strong CD4+ T-cell responses were induced; CD8+ responses were present but less prominent.	Env-specific binding antibodies, antibody-dependent functions	(146)
ASCENT/HVTN118/HPX2003	NTC02935686	Ad26.Mos4 HIV, Clade C GP140 plus adjuvant, Clade C GP140/mosaic GP140 plus Adjuvant, GP140 HIV Bivalent	Phase I	2017-2023	IFN-gamma ELISPOT and ICS (strong CD4+ toward Env, lower CD8+ toward Env compared to CD4+), higher CD8+ towards Gag, Pol, Central and effector memory CD4+ T cells	Env-specific IgG antibody, Nab against clade CMW965.26 and Clade B SF162.LS	(149)
TRAVERSE/HVTN117/HPX2004	NTC02788045	Ad26.mos.HIV, AD26.Mos4, HIV Clade C GP140	Phase I and II (1/2a)	2016-2022	IFN-gamma ELISPOT and Env-Specific CD4+ T cell, Gag, Pol specific CD8+ T cell	Clade C ENV Binding Antibody and Nab towards MW965.26	(151)
MOSAICO/HVTN 706	NCT03964415	AD26.Mos4.HIV, Clade C GP140/Mosaic GP140 Adjuvanted aluminum Phosphate AD26.mos1 Gag/Pol AD26.most2 Gag/Pol Ad26.Mos1. Env AD26.Most25.Env	Phase III	2019-2023	NA	NA, No efficacy	(153)
HIV-CORE 0052	NTC04586673	ChAdOx1+tHIVConsv1, MVA.tHIVConsv3 and MVA.tHIV cons4	Phase I	2021-2022	IFN-gamma ELISPOT: HIVconsvX-Specific T cell, polyfunctional CD8+ T cells, Terminal effector memory, translational memory, and central memory CD4+ T cell, effector and terminally differentiated CD8+ T cell. *In vitro* virus inhibition by CD8+ T cell effector	NA	(158)
HIV-CORE006	NTC04553016	ChAdOx1.tHIVConsv1, MVA.tHIVConsv3 and MVA.tHIVConsv 4	Phase I	2021-2022	IFN-gamma ELISPOT, polyfunctional CD4+, CD8+ T cell (IFN-gamma, TNF-alpha, IL-2, MIP-1 alpha), Inhibitor HIVconsuX-Specific T cell on four major global clades	NA	(160)
AELIX-002	NTC03204617	DNA.HII, MVA.HTI, ChAd OX1.HTI	Phase I	2017-2021	IFN-gamma ELISPOT, ICS: CD4+, CD8+ (IFN-gamma-GzmB/TNF-alpha, IL2, GzmB)	NA	(162)
AELIX-003	NTC04364035	MVA.HTI, ChAdOX1.HTI + Drug (GS-9620, TLR7 agonist)	Phase II	2020-2022	HTI-specific T cell ELISPOT-IFN-gamma and polyfunctional CD4+ and CD8+ T cell	NA	(164)
ADd5HVR48.ENVA.01/IDCAVD-002	NCT00695877	Ad5.ENVA.48 HIV-1	Phase I	2009-2016	IFN-gamma ELISPOT	EnvA-specific binding antibody, No NAb against a panel of titer 1 virus	(170)

The STEP (HVTN 502) (117) and Phambili (HVTN 503) (118) phase IIb trials represented pivotal moments in HIV vaccine research. The STEP trial (119–122) conducted across the Americas, Australia, and the Caribbean, tested a Merck HAdV-5vaccine encoding *gag*, *pol*, and *nef* from HIV-1 subtype B in men who have sex with men and at-risk women. Designed to assess both infection prevention and viral load reduction, the trial was halted in 2007 after interim analysis showed no efficacy. Alarmingly, uncircumcised, HAdV-5-seropositive men who received the vaccine had higher HIV acquisition rates than placebo recipients, particularly within the first 18 months, while initial apparent protection in circumcised, HAdV-5-seronegative men waned over time. It is worth mentioning that it was a *post-hoc* analysis while no significant improvement was achieved according to the pre-planned analyses.

The Phambili trial (123–126) in South Africa tested the same vaccine (clade B) in a clade C setting. It stopped concurrently with STEP, and participants were unblinded and monitored for long-term outcomes. Although differences between vaccine and placebo groups were not statistically significant, a trend toward higher infection rates in vaccine recipients persisted, with an adjusted hazard ratio of 1.25 after 25 months—reinforcing the concerns raised by STEP.

The STEP and Phambili trials highlighted the inherent limitations and risks of first-generation HAdV-5-based HIV vaccines. Although these vaccines elicited potent T cell responses to *gag*, *pol*, and *nef* antigens, this immunogenicity alone proved insufficient to prevent infection or control viremia. Moreover, the studies revealed that vector-induced immune modulation could influence susceptibility, particularly among individuals with pre-existing HAdV-5 immunity or other cofactors such as lack of circumcision. Viral sequence analyses from participants who became infected demonstrated selection at *gag*, *pol*, and *nef* epitopes, indicating that the vaccines exerted measurable immune pressure despite failing to confer protection.

In some subgroups, HAdV-5vaccination was associated with increased HIV-1 acquisition risk. Proposed explanations for this failure include inadequate magnitude or quality of cellular responses, absence of Env-specific antibodies, and limited coverage of globally diverse HIV-1 strains. These results suggest that while HAdV-5vectors effectively primed immune responses, the virus’s capacity for rapid escape from CD8^+^ T cell–mediated control rendered these responses ultimately ineffective, consistent with prior preclinical observations. Collectively, the STEP and Phambili outcomes prompted a strategic shift toward alternative adenoviral platforms, including serotypes with lower global prevalence, hexon-chimeric constructs, and non-human adenoviruses, designed to circumvent pre-existing immunity and elicit more protective and durable immune responses (127).

#### Alternatives for human adenovirus 5

Following the disappointing outcomes of the STEP and Phambili trials with HAdV-5-based vaccines, research attention turned to next-generation adenoviral vectors engineered to circumvent HAdV-5-associated limitations, specifically the pre-existing immunity. This effort emphasized alternative human serotypes with lower global seroprevalence, such as Ad26 and Ad35, and chimeric constructs like Ad5 HVR48, which replace hypervariable regions of HAdV-5hexon proteins to minimize neutralization by pre-existing antibodies. In parallel, non-human adenoviruses—particularly those originating from chimpanzees and other primates—were developed to further reduce cross-reactivity with HAdV-5immunity while maintaining potent immunogenicity in preclinical models (128). Beyond antigenic distinctness, these newer vectors also exhibit biological divergence from HAdV-5 (129): many preferentially engage CD46 rather than CAR for cell entry, display modified tissue tropism with reduced hepatic tropism, and interact differently with dendritic cells. Such variations influence innate activation patterns and ultimately modulate the magnitude, quality, and persistence of adaptive immune responses (130). Together, these innovations aim to retain the immunological potency of adenoviral vaccination while overcoming the immunological barriers that hindered HAdV-5-based approaches.

#### Human adenoviral 35 based HIV-1 vaccines

Ad35 vectors have relatively low global seroprevalence and distinct cellular tropism compared with HAdV-5 (131), providing the potential to bypass preexisting immunity and confer protection, as demonstrated in non-human primate studies (132). Preclinical data indicate that Ad35 is not cross-neutralized by HAdV-5-specific antibodies, allowing Ad35-vectored vaccines to maintain their immunogenicity even in the presence of preexisting HAdV-5neutralizing antibodies (133). As a Group B adenovirus, Ad35 utilizes CD46—an inhibitory complement receptor present on all nucleated human cells—for entry. This receptor specificity has constrained preclinical evaluation of rAd35 vectors, since most animal models do not express endogenous CD46 (134).

The VRC 012 study (135) examined heterologous vector combinations in a phase I trial, priming with rAd35-EnvA (VRC-HIVADV027-00-VP) and boosting with HAdV-5-EnvA (VRC-HIVADV038-00-VP). The rAd35-EnvA vaccine was immunogenic and safe with mild side effects however the observed aPTT abnormalities reflected an assay-related artifact rather than a clinical safety concern, arising from a transient, vaccine-induced anti-phospholipid antibody response—an effect previously documented in other adenoviral vector vaccine studies. When used in a reciprocal prime-boost regimen with HAdV-5-EnvA, it effectively primed and boosted antibody responses. However, in seronegative healthy volunteers, a three-month interval regimen did not significantly enhance T-cell responses beyond those induced by a single dose of HAdV-5 (136).

The IAVI B003/IPCAVD-004 trial (137) tested Ad26.EnvA and Ad35.Env vectors encoding clade A Env antigens in diverse populations across the U.S. and Africa in homologous or heterologous schedule. All regimens were well tolerated and immunogenic, with heterologous Ad26–Ad35 vaccination eliciting stronger antibody titers than homologous or reverse sequences. T cell responses were moderate but consistent across groups (138).

The HVTN 083 trial (139) evaluated the safety and immunogenicity of a heterologous-insert, prime-boost HIV vaccine regimen incorporating inserts from multiple HIV-1 subtypes delivered via combinations of adenovirus vectors (HAdV-5or Ad35) and HIV-1 envelope (Env) gene inserts (clade A or B). T-cell responses elicited by both heterologous and homologous insert regimens targeted a similar overall number of epitopes. However, heterologous insert regimens generated significantly more shared epitopes between EnvA and EnvB and induced responses against epitopes with greater evolutionary conservation, providing higher coverage among responders. Overall, heterologous vector regimens recognized a larger number of totals, EnvA, and EnvB epitopes compared with homologous vector regimens (140).

Ad35-vectored HIV vaccines have been shown to be safe and immunogenic in humans. As a prime in heterologous regimens with HAdV-5or Ad26 boosts, Ad35 effectively elicited antibody responses with modest T cell responses and the results offer important insights into prime-boost strategies that can reshape the design of effective HIV vaccines.

#### rAd26-based HIV-1 vaccine candidates

Ad26-based vectors have consistently demonstrated a favorable safety profile and the ability to elicit both antibody-mediated and T cell responses, although not all constructs have achieved potent immunogenicity in clinical settings (141). In addition, studies in rhesus macaques have shown that when Ad26-based vaccines are paired with secondary vectors—such as MVA or Ad35—the resulting heterologous regimens can reduce susceptibility to SIVmac251 infection and improve post-challenge viral containment (132).

The IPCAVD 001 trial (142) marked the first human evaluation of an Ad26-based HIV vaccine. Participants received three doses of Ad26.ENVA.01 or placebo. The vaccine induced both humoral and cellular responses, including binding antibodies, ADCC activity, and CD4^+^/CD8^+^ T cell responses. Although antibody responses were non-neutralizing, an important finding was that intramuscular delivery could elicit strong mucosal immune responses. Interestingly, Env-specific binding antibodies were dominated by the IgG1 isotype (>90%), with only modest levels of IgG3 and no detectable serum IgA. Because IgG1 and IgG3 efficiently engage specific Fc receptors, the antibody response elicited by Ad26.ENVA.01 is likely well suited for recruiting innate effector mechanisms. Notably, this IgG1/IgG3-dominated pattern mirrors the profile observed in RV144 (143, 144), whereas VAX003 (monovalent subtype B and bivalent subtype B/E (CRF01_AE) recombinant glycoprotein 120) instead generated elevated IgG2 and IgG4 responses (145). Building on this platform, the Imbokodo phase IIb trial (146) tested a Ad26.Mos4.HIV vaccine combined with a gp140 protein (Clade C) boost in young women at high HIV risk in sub-Saharan Africa. This vaccine platform demonstrated exceptionally strong protection on a per-exposure basis in nonhuman primate studies (SHIV-SF162P3/rhesus macaque model) (147). Despite the proven efficacy in NHP model, ultimately this trial terminated early due to lack of efficacy. This trial showed that the heterologous regimen was immunogenic—eliciting Env-specific binding antibodies, antibody-dependent functions, and antigen-specific CD4+ and CD8+ T-cell responses — but these responses did not translate into statistically significant protection against HIV acquisition. However, the vaccine was well tolerated with no serious adverse events and despite its failure, Imbokodo confirmed Ad26’s strong safety profile and ability to generate broad immune responses. Multiple factors may have contributed to the failure of protection in this trial, including intense exposure risk, broad viral diversity, host cofactors that heightened susceptibility, and immune responses that were ultimately inadequate (148).

To further evaluate the potential of Ad26.Mos4.HIV, the ASCENT trial (149), a phase IIa study in Kenya, Rwanda, and the U.S., was assessed efficacy of this candidate with either bivalent Clade C/mosaic gp140 or Clade C gp140 (both alum-adjuvanted) versus placebo. Among healthy adults, vaccination produced potent humoral and cellular immune responses including binding antibodies to Mos1 gp140 and vaccine-elicited CD4+/CD8+ T cells responses. Interestingly, Individuals who mounted the most pronounced CD4^+^ T-cell responses to Env also generated the highest Mos1 gp140–binding antibody levels, underscoring the contribution of T-cell help to potent antibody development. In contrast, CD8^+^ T-cell activity against Env was comparatively modest and rose more slowly across both vaccine regimens (150).

The TRAVERSE trial (149, 151), a randomized phase I/II a study across the U.S. and Rwanda, compared trivalent (Ad26.Mos.HIV) and tetravalent (Ad26.Mos4.HIV) regimens with clade C gp140 protein boosts. Both were well tolerated, with mostly mild reactogenicity. By the second dose, every per-protocol participant generated clade C Env–specific binding antibodies, and responses were consistently stronger in those receiving the tetravalent formulation. This broader vaccine also elicited higher cross-clade antibody reactivity, more potent IFNγ ELISPOT activity, and increased frequencies of Env-specific CD4^+^ T cells following subsequent boosts. Importantly, pre-existing Ad26 immunity did not dampen vaccine performance (152).

The MOSAICO study (153) assessed the efficacy of a heterologous vaccine regimen combining Ad26.Mos4.HIV with aluminum phosphate–adjuvanted Clade C and Mosaic gp140 proteins to prevent HIV-1 infection in HIV-negative cisgender men and transgender individuals engaging in sexual activity with cisgender men and/or transgender partners. According to the Mosaico DSMB review of current data, the vaccine regimen showed no evidence of protection against HIV and was unlikely to achieve its primary efficacy endpoint. No safety concerns were observed with the regimen.

Overall, Ad26-based vaccine studies, including HVTN705 (Imbokodo) and HVTN706 (Mosaico), demonstrated potent immunogenicity in humans and protective effects in nonhuman primates, yet were stopped after primary analyses showed no efficacy. These trials highlight both the potential and the constraints of Ad26 platforms: they are safe, versatile, and capable of supporting approaches like mosaic antigen design and heterologous boosting while they do not elicit the required immunogenicity for protection. Beyond the outcomes, the trials offer critical lessons for HIV vaccine development. Imbokodo, in particular, set a standard for ethical trial conduct by engaging high-risk, underrepresented communities, collaborating with local stakeholders, and promoting additional prevention measures such as PrEP, providing a framework for future HIV-1 prevention efforts (154).

#### Chimpanzee adenoviral–based HIV-1 vaccine candidates in heterologous regimes

Chimpanzee-derived adenoviral vectors, particularly ChAd3 and ChAd63, have emerged as promising HIV-1 vaccine platforms because of their low seroprevalence in humans across Africa, the Americas, and India (155). This feature reduces the barrier of pre-existing vector immunity that limits human adenoviruses such as HAdV-5 (156). When used to deliver HIV or SIV gag transgenes, both ChAd3 and ChAd63 induced immunogenicity equivalent to HAdV-5and outperformed other recombinant human adenoviruses in eliciting potent T cell responses (155, 157). Building on these properties, several clinical trials have evaluated ChAd-based regimens focused on highly conserved regions of HIV-1 mainly used as T-cell vaccines, often in prime–boost regimens with MVA or DNA. These viral vectors are also used as therapeutics HIV-1 along with MVA and DNA platforms.

The HIV-CORE 005.2 trial (158) was a first-in-human, open-label, dose-escalation study at the University of Oxford evaluating the HIVconsvX T cell–focused vaccine platform. HIVconsvX comprises conserved regions from Gag and Pol, excluding Env to circumvent its variability, and is designed to elicit broad T cell responses against stable viral elements. Participants received a prime–boost regimen (C1C62–M3M4) using ChAdOx1-vectored HIVconsv1 and HIVconsv62 for priming, followed by MVA-vectored HIVconsv3 and HIVconsv4 for boosting. Vaccines were well tolerated, with no serious adverse events and only expected local and systemic reactogenicity. All participants developed detectable T cell responses by IFN-gamma ELISPOT, which, although waning 7.4-fold by day 140, retained proliferative capacity, polyfunctionality, recognition of variants, and the ability to inhibit HIV-1 from clades A–D. These results highlight ChAdOx1–MVA-vectored conserved mosaic HIVconsvX candidate T-cell vaccine’s potential to induce broad, functional, cross-clade T cell immunity (159).

The HIV-CORE 006 trial (160) extended the previous trial in a randomized, double-blind, placebo-controlled study across Uganda, Kenya, and Zambia using the C1–M3M4 regimen (ChAdOx1.tHIVconsv1 prime, MVA.tHIVconsv3/MVA.HIVconsv4 boosts). The aim was to induce effective cytotoxic T lymphocytes (CTL) against HIV-1. Generating potent HIV-1–specific cytotoxic T cells is critical, as they can synergize with broadly neutralizing antibodies to enhance protection and contribute to curative strategies. The vaccine was safe and well tolerated. Immunogenicity analyses showed that 99% of participants mounted detectable HIVconsvX-specific T cell responses. Across the 40-week follow-up period, CD8^+^ T cells showed a marked shift toward a T effector cell-dominant profile, accompanied by declines in both T effector memory cells (TEM) and transitional memory T cell (TTM) subsets. In contrast, CD4^+^ T cells displayed the opposite pattern, with TTM cells emerging as the most expanded population and TEM cells showing the greatest reduction. Despite the decline in responses over the time, the immunity retained functional competence—including proliferation, polyfunctionality, and inhibition of multiple HIV-1 clades—demonstrating safety, durability, and cross-clade functionality of HIVconsvX vaccines in African populations (161).

As an effort to design therapeutic HIV-1 vaccines, AELIX Therapeutics elicited an innovative T-cell–based HIV vaccine candidate aimed at enabling individuals living with HIV to maintain control of viral replication without continuous antiretroviral therapy. The vaccine’s core component, known as the HIVACAT T cell immunogen (HTI), incorporates strategically selected regions of the virus that are most susceptible to immune targeting and designed to induce cellular responses targeting HIV regions linked to viral control in humans. The AELIX-002 trial (162) administered a combination of DNA.HTI, MVA.HTI, and ChAdOx1.HTI vaccines to 45 early-ART–treated individuals to assess safety, immunogenicity, and effects on viral rebound during ATI. Vaccines were well tolerated and elicited broad, polyfunctional CD4^+^ and CD8^+^ T-cell responses. Notably, HTI immunization generated robust and durable GzmB-producing CD8^+^ T cells and enhanced their capacity to suppress replication of CCR5- and CXCR4-tropic viruses, as well as autologous HIV strains spanning a wide range of replicative fitness. Vaccine performance was tested in a monitored antiretroviral treatment interruption (ATI). All participants experienced viral rebound during ATI, although stronger HTI-specific T-cell responses correlated with longer time off ART, suggesting potential utility in combination cure strategies despite limited efficacy in preventing rebound (163). Building on these results, the AELIX-003 trial (164) evaluated ChAdOx1.HTI and MVA.HTI of AELIX Therapeutics combined with Gilead´s Toll-Like Receptor 7 (TLR7) agonist GS-986, an analogue of vesatolimod, in early-treated, virally suppressed men. Vaccination was well tolerated and induced strong, broad HTI-specific T-cell responses. While all participants experienced viral rebound during a 24-week ATI, higher HTI-specific T-cell levels were associated with longer time off ART, supporting the potential of HTI vaccines with vesatolimod as a safe, T-cell–focused strategy in HIV cure research (165).

Clinical evaluation of chimpanzee adenoviral (ChAd) vector–based HIV-1 vaccines, including the AELIX (HTI) and HIVconsvX programs, has shown that these platforms are safe, well tolerated, and capable of eliciting strong, polyfunctional T-cell responses targeting conserved viral regions. Trials such as AELIX-002, -003, and HIV-CORE 005/006 provide evidence that therapeutic vaccination with ChAdOx1 followed by an MVA boost—alone or combined with the TLR7 agonist vesatolimod—can enhance immune control of viral replication during analytical treatment interruption, particularly in participants lacking protective HLA alleles or mounting strong vaccine responses. These studies highlight key protective T-cell features, including proliferative capacity, polyfunctionality, recognition of founder viruses across conserved epitopes, and both high- and low-affinity clonotype contributions that support viral suppression through cross-reactivity, infected-cell killing, and antiviral mediator production. While most analyses were limited to PBMCs rather than lymphoid or mucosal tissues—critical sites for HIV-1 replication—the findings support HTI vaccines as a T-cell–priming bacKbpone for combination cure strategies, which may be further potentiated with immunomodulators, bNAbs, B-cell vaccines, or alternative vectors. Together, these results underscore the promise of ChAd-based prime–boost regimens as versatile platforms for both preventive and therapeutic HIV vaccine development, warranting further evaluation in larger clinical trials.

#### Hexon-chimeric adenoviral based HIV-1 vaccine candidates

The adenoviral hexon protein—the most abundant capsid component and major target of host immunity—is a key determinant of anti-vector responses that can significantly limit the efficacy of adenovirus (AdV)–based vaccines and gene therapies (166). Each viral capsid contains 720 hexon monomers with a conserved core and seven hypervariable regions (HVR1–HVR7) forming surface-exposed loops that elicit strong neutralizing antibody responses (167). The length and orientation of these HVRs vary by serotype; in HAdV-5, for instance, HVR1 spans 44 amino acids. Structural analyses show that HVR1, HVR5, and HVR7 are prominently exposed at the hexon apex and thus highly accessible to antibodies, while HVR2–HVR6 lie closer to the base. All HVRs harbor type-specific epitopes that mediate serotype-specific immune recognition. To circumvent pre-existing immunity to common serotypes such as HAdV-5, hexon chimerization—replacing exposed HVRs with those from alternative serotypes—has been employed to evade neutralization while maintaining vector functionality, thereby enhancing transgene delivery and immunogenicity (168, 169).

The first-in-human trial of the hexon-chimeric vector Ad5HVR48.ENVA.01/IPCAVD-002 (170) evaluated Ad5HVR48.EnvA.01, an HAdV-5vector incorporating Ad48 hexon HVRs and encoding the HIV-1 EnvA antigen. In this randomized, double-blind, placebo-controlled phase I dose-escalation study, 48 HIV-uninfected, HAdV-5/Ad48-seronegative adults received either a single dose or three doses. The vaccine was well tolerated, with only mild, transient reactogenicity at the highest dose and no serious adverse events. It elicited durable EnvA-specific IgG responses that persisted through week 52, alongside strong EnvA-specific IFN-γ ELISpot activity. Additionally, neutralizing antibody responses were stronger following Ad48 vaccination compared with HAdV-5, indicating that human Ad-specific nAbs predominantly—but not solely—target the hexon hypervariable regions (171).

These findings establish, for the first time, the safety and immunogenicity of the recombinant Ad5HVR48.ENVA.01 vaccine in humans. The vaccine consistently induced Env-specific humoral and cellular responses across a 100-fold dose range, with minimal reactogenicity even at the highest dose. Further studies are required to assess whether preexisting immunity to HAdV-5or Ad48 influences observed safety or immunogenicity. As a biologically distinct vector from parental HAdV-5, the chimeric Ad5HVR48 warrants continued investigation as a platform for HIV and other pathogens.

To summarize, despite being responsible only for mild human illnesses, adenoviral platforms face a major drawback: many individuals harbor pre-existing antibodies that rapidly neutralize the vector. This early immune interception reduces antigen expression and may even heighten susceptibility to HIV infection in certain contexts. Yet, in comparison with other viral delivery systems, adenoviruses continue to stand out because they generate remarkably strong and long-lived T-cell responses—an immunological feature considered essential for durable HIV control. Although widespread seropositivity has historically limited their effectiveness, extensive research has produced multiple approaches to bypass this obstacle. For example, alternative serotypes capable of replicating in the hostcan be administered orally offering a potential route around pre-existing immunity. Mucosal administration routes, including intranasal or oral delivery, have also yielded superior protective immunity in animal studies and may better engage front-line defenses at sites relevant to HIV transmission (172).

Another strategy under consideration involves modifying adenoviral vectors to minimize CD4+ T-cell stimulation, which could mitigate concerns about increased HIV acquisition risk; however, restricting CD4+ T-cell involvement might weaken the support required for the development and persistence of both CD8+ T-cell responses and antibody production.

### Poxviruses as vaccine platform for HIV-1

#### Poxvirus biology

Poxviruses have long been explored as viral vectors for vaccine development, against different viral pathogens including HIV-1, because of their favorable biological properties. They are large double-stranded DNA viruses with genomes ranging from 130 to 300 kilobase pairs (Kbp) (173). Several features make them attractive platforms: they do not integrate into the host genome (174), are strongly immunogenic (175), can accommodate a large capacity for inserting genes of interest (GOIs) (176), and support efficient gene expression driven by different poxviral promoters (177). The Poxviridae family is divided into two subfamilies: *Chordopoxviridae* (poxviruses of vertebrates) and *Entomopoxvirinae*. Within *Chordopoxviridae*, the genera *Orthopoxvirus* and *Avipoxvirus* have been most extensively engineered as recombinant vaccine vectors (178). In designing effective vaccines based on these viruses, both the choice of promoter and GOI optimization are critical (179). For example, anti-HIV-1 Env-specific antibody levels were significantly higher when using the SFJ1–10 promoter compared with the widely used p7.5 promoter while canarypox construct expressing HIV-1 JR-CSF env gene in C57BL/6J mice (180).

Poxviral vectors are particularly effective at inducing CD4^+^ T cell responses, as shown in both preclinical and clinical studies (181). Several strains have been developed as candidate HIV-1 vaccine vectors (**Table 3**), including Modified Vaccinia Ankara (MVA), canarypox virus (ALVAC), replication-restricted New York vaccinia virus (NYVAC), and a replication-competent derivative, NYVAC-KC (182). Each possesses distinct biological properties that influence their safety, immunogenicity, and suitability for vaccine applications, and each is discussed in detail below.

**Table 3:** Poxviral based HIV-1 vaccines.

Trial	Clinical trial ID	Vaccine platform	Phase	Start- end	Cellular response	Humoral response	Ref
HVTN 065	NTC00301184	pGA/JS7 DNA, MVA/HIV62	Phase I	2006-2008	ICS: Gag and Env specific CD4+/CD8+ T cell response (IFN-gamma, IL2)	Env specific binding Antibodies	(198)
HVTN 205	NTC00820846	pGA/JS7 DNA, MVA/HIV62	Phase II	2009-2014	Specific CD4+ and CD8+ T cell, polyfunctional CD4+ and CD8+ (IFN-gamma, IL2, TNF-alpha), CD8+ polyfunctional> CD4+ polyfunctional	Neutralization antibodies, Specific binding antibody GP41-IgG>GP120-IgG	(199)
HVTN 094	NTC01571960	GEO-DO3 DNA, MVA/HIV62B	Phase I	2012-2016	ICS: CD4+> CD8+ cells (IL-2/IFN-gamma), both CD4+ and CD8+ mainly targeted Gag	Env-GP140 and GP41 binding IgG, No IgA response, ADCC response	(202)
TBC-M4	NCT00902824	MVA expressing enc, gag, RT, rev, tat, and nef subtype C	Phase I	2008-2010	IFN-gamma ELISPOT and ICS: CD4+ and CD8+ (IFN-gamma)	No neutralization antibody, HIV-specific binding antibody	(205)
RisVac02	NTC00679497	MVA-B	Phase I	2008-2010	ELISPOT IFN-gamma	binding antibody to GP160, neutralizing antibodies in 33% of vaccines	(207)
RisVac03	NTC01571466	MVA clade B expressing Bx089p120 and IIIB GagPolNef	Phase I	2011-2013	T cell response to Gag. No effect on rebound of plasma viral load after interruption of cART	NA	(208)
CR100965	NTC02218125	MVA.Mosaic (MVA.Mos1 and MVA.mos2) expressing gag/pol/env	Phase I	2014-2015	IFN-gamma ELISPOT and ICS modest CD4+/CD8+ T cells (IL2 and IFN-gamma)	EnvA specific binding antibody, No neutralizing antibodies	(210)
RV144	NTC00223080	ALVAC-HIV (vCP1521), AIDSVAX gp120 B/E	Phase III	2003-2009	ICS: modest CD8+ cells (IFN-gamma/IL-2), Robust CD4+ T cells to Env V2 loop [31.2% efficacy at 42 months, 60% efficacy at 12 months]	No neutralizing antibodies to V1V2, high ADCC, HIV-1 specific IgG3. and Binding antibody to V2, V3 and C5 region in GP120	(218
RV305	NTC01435135	ALVAC-HIV, AIDSVAXB/E	Phase II	2012-2021	Polyfunctional Gag-Env and V2 specific CD4+ and CD8+ T cells. ICS markers:CD154, CD107, IL-2, IL-4, IFN-gamma and TNF-alpha	Binding antibodies towards V2 and V3 and CD4i, ADCP, ADCC	(225)
EV01	NA	NYVAC-C	Phase I	2003-2004	ELISPOT IFN-gamma against env, gag, pol and nef, ICS: polyfunctional CD4+, CD8+ T cells (IFN-gamma and IL-2)	IgG response to CN54 GP140 antigen	(238)
EV02	N/A	DNA-C prime, NYVAC-C boost	Phase I	2005-N/A	ELISPOT IFN-gamma, ICS CD4+ and CD8+ (IL-2, IFN-gamma and TNF-alpha)	GP140-specific IgG binding antibody	(240)
HVTN 078	NTC00961883	NYVAC-B and rAd5	Phase I	2009-2013	ICS: CD4+ and CD8+ (IL-2, IFN-gamma)	Env-specific binding antibody, bNab against tier 1 viruses, V1-V2 antibody lower than RV144	(243)
HVTN 096	NCT01799954	DNA-HIV-PT123, NYVAC-HIV-PT1, NYVAC-HIV-PT4, AIDSVAX B/E	Phase I	2012-2014	ICS gag and Env specific CD4+ and CD8+ (IL-2, IFN-gamma)	Binding IgG, Nab towards tier 1 and ADCC	(245)

#### Modified vaccinia Ankara biology

Modified Vaccinia Ankara (MVA) is a replication-deficient, highly attenuated poxvirus vector derived from the chorioallantois vaccinia virus Ankara (CVA) through more than 570 serial passages in primary chicken embryo fibroblasts (CEF) (183, 184). This extensive attenuation resulted in a 178 Kbp genome containing six major deletions and numerous smaller mutations affecting 122 of its 195 open reading frames, eliminating approximately 30 Kbp of genetic material—including many virulence and immune evasion genes (185). Transgenes are commonly inserted at sites such as the thymidine kinase and hemagglutinin loci or within Deletions II, III, and V, which enable stable foreign gene expression without impairing vector stability or replication in permissive cells (185, 186). MVA offers several advantages as a vaccine vector: it is replication-incompetent in most mammalian cells, can be handled under BSL-1 conditions in many countries, and retains strong immunogenicity despite transient antigen expression (187). It promotes antigen presentation through both endogenous and cross-presentation MHC class I and II pathways, lacks soluble viral inhibitors of interferon and TNF signaling, and does not block NF-κB activation—enhancing recruitment and activation of immune cells (188, 189). With a transgene capacity of approximately 10 Kbp, MVA has demonstrated an excellent safety profile in clinical trials, though residual immunity from historical smallpox vaccination campaigns remains a potential drawback (190).

Promoter selection and expression kinetics are also crucial for optimizing recombinant MVA (rMVA) vaccines. Poxviral transcription proceeds through early, intermediate, and late phases, with intermediate-stage expression yielding the most potent CD4^+^ and CD8+ T cell responses (179). The promoter spacer length can modulate immunogenicity, whereas promoter timing (early vs. late) has minimal effect on antibody production against the encoded antigen. rMVA-mediated antigen expression typically peaks around 6 hours post-infection, declines by 24 hours, and becomes undetectable within 48 hours (191). Although rMVA vectors are safe and immunogenic, responses are often modest in magnitude, breadth, and durability. To address this, next-generation MVA platforms aim to enhance both humoral and cellular immunity by optimizing the viral bacKbpone, modifying or codon-optimizing antigen sequences, employing stronger or antigen-specific promoters, and incorporating tailored prime–boost regimens (192, 193). These approaches seek to improve the magnitude, polyfunctionality, and persistence of responses while retaining the hallmark safety of MVA.

### Modified vaccinia Ankara based HIV-1 vaccines

Across numerous preclinical and clinical studies, including trials in both healthy volunteers and HIV-1–infected cohorts, MVA-based vaccines have consistently proven safe and well tolerated, even when given repeatedly or in heterologous prime–boost regimens (194–196). MVA constructs expressing HIV-1 antigens reliably induce cellular and humoral responses despite pre-existing immunity against pox viruses, though homologous MVA-only boosts often produce modest magnitude, limited breadth, and reduced durability, constraining its potential as a standalone platform (197). Several approaches have been explored to improve the immunogenicity and efficacy of MVA-based vaccine candidates, including optimization of the MVA vector and the inserted heterologous antigens, refinement of prime–boost immunization strategies, and the use of stronger viral promoters (198).

DNA–MVA prime-boost vaccination has shown strong immunogenic potential against HIV-1 in preclinical studies and clinical trials. First−in−human studies of T−cell–focused HIV−1 genetic vaccines evaluated a heterologous pTHr.HIVA DNA prime followed by MVA.HIVA boost in several hundred volunteers in Europe and Africa, including both uninfected individuals and people living with HIV−1. In larger Phase I trials, HIV−1−specific T−cell responses were detected in a minority of healthy vaccinees (generally <15%), whereas smaller studies with deeper immunoprofiling provided clearer mechanistic insight. Overall, pTHr.HIVA primed reproducible but low−magnitude, predominantly CD4^+^ responses, while MVA.HIVA consistently boosted both CD4^+^ and CD8^+^ T cells, with particularly strong amplification in HIV−1−infected participants on ART. These data support ongoing efforts to improve DNA priming while underscoring MVA as an effective boosting vector for cellular−immunity–based vaccine platforms (199–201).

The GeoVax`s HIV vaccine strategy evaluated the safety and immunogenicity of combination of a DNA prime, pGA2/JS7 produces non-infectious virus-like particles (VLPs), and encodes HIV-1HXB-2 Gag, HIV-1BH10 PR and RT, and Env, Tat, Rev, and Vpu derived from a recombinant of the HXB-2 and ADA strains of HIV-1 with a recombinant MVA/HIV62B encodes HIV-1 Gag, PR, RT, and Env from the same sequences as JS7 and also produces noninfectious VLP boost. Phase I (HVTN 065) (202) and Phase IIa (HVTN 205) (203) studies confirmed both approaches were safe and well tolerated. Across the HVTN 065 trial, reactogenicity was generally mild to moderate, and the pattern of immune activation varied sharply by regimen. Participants receiving the DDMM regime (2 doses of JS7 DNA vaccine and 2 doses of MVA/HIV62B) showed the strongest CD4^+^ and CD8^+^ T-cell responses, whereas those vaccinated exclusively with MMM regime (3 doses of MVA/HIV62B) exhibited the weakest cellular immunity. The opposite trend was observed for antibody outcomes: the MMM regimen produced the most strong Env-specific binding and neutralizing antibody responses, with the DMM schedule landing between the two extremes. Notably, MVA62 demonstrated a favorable safety profile and triggered distinct cellular and humoral response profiles depending on whether it was delivered as a standalone vector or paired with the JS7 DNA prime (204).

Findings from HVTN 205 reinforced these trends. At peak immunogenicity, binding antibodies to Env were detected in 93.2% of individuals primed with DDMM and 98.4% of those receiving MMM. These antibody responses were consistently stronger against gp41 than gp120. In both vaccine strategies, CD4^+^ T-cell responses dominated over CD8^+^ T-cell responses, with Gag emerging as the primary antigenic target. More than 70% of responsive T cells—across both CD4^+^ and CD8^+^ compartments—exhibited polyfunctionality, secreting two or three of the cytokines assessed (IFN-γ, IL-2, TNF-α, and granzyme B) (205). In another study (HVTN 094) (206). The safety of two injections of GEO-D03 DNA priming vaccine co-expresses HIV-1 clade B proteins, Gag, protease, RT, gp160 Env, Tat, Vpu and Rev, as non-infectious VLPs and human GM-CSF followed by either two or three boosting injections of MVA/HIV62B (MVA62B) vaccine were evaluated. This DNA/MVA prime–boost regimen generated durable and functional humoral responses, including ADCC activity, high antibody avidity, and strong Env-specific IgG1 and IgG3 binding to the immunodominant gp41 region. Analyses of cytokine-producing T cells revealed that the vaccine more readily activated CD4^+^ T cells, with IL-2 and IFN-γ responses occurring at greater frequencies than in the CD8^+^ compartment. Most of the detectable activity in both subsets targeted Gag, while Env-specific responses were observed less frequently and Pol-directed responses were largely absent (207). Broader antigen coverage with multi-gene MVA constructs can further enhance immunogenicity (208).

For instance, two TBC-M4 and MVA-B vaccines underscore the promise of Modified Vaccinia Ankara (MVA) as a safe and immunogenic vector platform for HIV vaccine development. TBC-M4 (209) is an MVA-based recombinant vaccine candidate developed by Therion Biologics Corporation (Cambridge, MA). This construct incorporates a panel of HIV-1 subtype C genes of Indian origin, including env, gag, the reverse transcriptase (RT) gene, as well as the regulatory elements rev, tat, and nef. In a phase I dose-escalation trial, recombinant MVA-TBC-M4 showed good tolerability, and IFN-γ ELISPOT assays revealed antigen-specific responses concentrated on Env and Gag, increasing with higher doses. As expected for this construct, neutralizing antibodies to HIV-1 subtype C were not detected. Together, these data suggest that TBC-M4 may be better suited for inclusion within a heterologous prime–boost strategy rather than used alone (210).

MVA-B, which encodes gp120 and a Gag-Pol-Nef fusion from subtype B, has shown strong immunogenicity across some trials like RisVac02 (211) and RisVac03 (212). RisVac02, a phase-I randomized, double-blind study with placebo controls, was carried out to evaluate how a recombinant MVA platform encoding clade B HIV-1 antigens perform in terms of safety and immune activation. The trial showed that the MVA-B construct demonstrated an excellent safety profile and was generally well tolerated, while also generating sustained cellular and humoral immunity in most participants—T-cell responses in roughly three-quarters of individuals and antibody responses in nearly all. These outcomes highlight the suitability of MVA-B for continued advancement in HIV-1 vaccine development (194).

To further assess this platform, the RisVac03 trial was conducted with or without a drug to reactivate latent HIV-1 (disulfiram). In chronically HIV-1–infected individuals, administration of MVA-B proved to be safe and led to an elevation of Gag-directed T-cell immunity. However, whether delivered alone or together with disulfiram, the regimen produced little measurable effect on the size of the latent reservoir or on post-cART viral rebound (213).

Mosaic immunogen strategies have been extended to MVA vectors to enhance cross-clade coverage. A first-in-human, randomized, double-blind, placebo-controlled trial (214) evaluated a MVA Mosaic (composed of MVA Mosaic 1 and MVA Mosaic 2 delivering complementary HIV-1 *gag/pol/env* inserts) in vaccine-naive adults and individuals previously immunized with Ad26.ENVA.01 four to six years earlier. The immunization regimen showed an excellent tolerability profile, with no serious adverse events attributed to the vaccine. In individuals without prior HIV-1 vaccination, one or two doses triggered broad cellular and antibody responses that recognized multiple clades. Among participants previously primed with Ad26.ENVA.01, a single booster was sufficient to re-engage Env-specific immunity in almost every case (84).

MVA-based HIV-1 vaccines have consistently demonstrated excellent safety and tolerability in both healthy and HIV-infected individuals, with very few vaccine-related serious adverse events reported (215).

These vectors are well suited for use as boosters in heterologous prime–boost regimens, such as DNA- or ChAd-priming followed by MVA boosting, where they enhance HIV-specific cellular immunity relative to single-platform strategies. In this context, MVA reliably induces broad, polyfunctional CD4^+^ and CD8^+^ T-cell responses and also generates strong binding antibody responses across multiple clades, with response magnitude influenced by the immunization regimen and prior vaccination history. Moreover, MVA vaccination can safely re-engage pre-existing immunity in previously vaccinated individuals, amplifying both antibody and T-cell responses. However, in chronically HIV-1–infected patients on ART, therapeutic vaccination with MVA increases Gag-specific T cells but has little measurable effect on the latent reservoir or post-treatment viral rebound, whether used alone or in combination with agents like disulfiram.

Despite the potent immunogenicity of MVA vectors, challenges remain in translating immune responses into protective efficacy. Pre-existing anti-vector immunity can influence boosting but does not prevent MVA from eliciting antigen-specific responses. The main bottleneck for efficacy lies in immunogen design—broader, conserved, or mosaic antigens are needed to generate neutralizing antibodies and functional T-cell responses capable of protection based on the immune response data. In addition, the immunogen design is highly limited by the lack of a clear immune correlation of protection. Durability and quality of immune responses, including polyfunctionality and localization, are critical factors, not just the magnitude of the response. These lessons underscore the importance of multi-component regimens, innovative antigen design, and the use of more sophisticated immune assays to identify immune correlates of protection in future HIV vaccine strategies to move beyond immunogenicity toward true clinical protection.

### Canarypox viral vector (ALVAC)

#### Canarypox viral vector (ALVAC) biology

Canarypox virus, a member of the Avipoxvirus subfamily, has a natural host range limited to avian cells. In mammalian cells it supports early and late gene expression but does not complete replication. ALVAC was derived from a canarypox virus clone after four rounds of plaque purification from a vaccine strain for canaries (216). In human studies, ALVAC is well tolerated with no evidence of pre-existing cross-reactive immunity, unlike some other vectors, but its immunogenicity remains limited, with cytotoxic CD8^+^ T-cell responses observed in fewer than 25% of volunteers (217, 218).

### ALVAC based HIV-1 vaccines

ALVAC as a replication-defective viral vector has been extensively studied in HIV-1 vaccine research, following platforms such as MVA, NYVAC, and fowl pox, and is noted for its strong safety profile and capacity to engage both humoral and cellular immunity (219). To overcome its low cytotoxic CD8^+^ T-cell responses, ALVAC engineered to express CD40 ligand (CD154) to enhance cellular immunity and CD8^+^ T-cell memory in both HIV-1–infected and uninfected individuals. This modified vector matures human monocyte-derived dendritic cells independently of TNF-α signaling, reduces apoptosis, and efficiently expands ex vivo cytotoxic T lymphocyte responses against Epstein-Barr virus in healthy donors and HIV-1 in infected individuals, even without CD4^+^ T-cell help (220). Multiple clinical trials have tested ALVAC-based constructs expressing various HIV-1 antigens, either as standalone vaccines or as priming agents in heterologous prime–boost regimens, often combined with subunit proteins or synthetic lipopeptides (217, 221, 222).

The RV144 trial (Thai HIV Vaccine Study) (223) established ALVAC as a cornerstone of HIV vaccine development. Among HIV-1 vaccine efficacy trials, only the Phase III RV144 study has demonstrated statistically significant protection, primarily during the initial months following completion of the immunization schedule. This regimen consisted of a recombinant canarypox vector (ALVAC-HIV, vCP1521) delivering an Env antigen, followed by two boosts with the recombinant gp120 subunit vaccine AIDSVAX B/E (224–229). Despite initial skepticism regarding the modest immunogenicity of ALVAC compared with other vector-based approaches, RV144 demonstrated safety and achieved 60% vaccine efficacy at 12 months, declining to 31% at 3.5 years (230). Importantly, the trial allowed for the first systematic study of correlations of protection, revealing that neutralizing antibodies against circulating Tier 2 strains were largely undetectable, suggesting that non-neutralizing antibody functions—such as high-avidity IgG binding to Env, antibody-dependent cellular cytotoxicity (ADCC), and phagocytosis (ADCP)—were primarily responsible for the observed protection. IgG antibodies targeting the V1V2 region correlated inversely with infection risk, while binding IgA antibodies showed a direct correlation with infection, highlighting the impact of antibody specificity. Genomic sieve analyses further identified specific V2 (positions 169 and 181) and V3 loop sites under immune pressure, emphasizing the importance of antibody quality and targeting in HIV prevention. Cellular immune responses in RV144 were modest relative to other poxvirus- or adenovirus-based vaccines. CD8^+^ T cell responses were detectable by cytotoxicity assays but were infrequently measurable ex vivo (<10%) in standard IFN-γ/IL-2 ICS assays, comparable to placebo recipients. In contrast, strong CD4^+^ T cell responses were observed, predominantly targeting the Env V2 loop, which includes the α4β7 integrin binding site critical for establishing viral synapses in gut-associated lymphoid tissue. *Post hoc* analyses revealed that Env-specific CD4^+^ T cell responses, particularly polyfunctional cells expressing CD154 and secreting IL-2, IL-4, IFN-γ, and TNF-α, inversely correlated with HIV acquisition (85).

RV305 (231), the follow-up to RV144 in Thailand, evaluated late boosting 6–8 years post-primary immunization with AIDSVAX B/E gp120, ALVAC-HIV, or both in three different clinical trials. First, all three trials demonstrate that late boosting can effectively recall memory B cells established by the primary vaccination series, enhancing antibody magnitude, specificity, and affinity maturation. Delayed boosts, even years after priming, increased responses against key epitopes like the CD4 binding site, suggesting that memory B cells remain responsive and can undergo further maturation. However, durability of these boosted responses was limited, and additional boosts showed diminishing returns, highlighting that timing and scheduling critically influence the quality and persistence of humoral immunity. Second, the format and delivery of immunogens significantly shape the quality of the antibody response. Viral vectors improved breadth, Fc-mediated effector functions, and durability compared to protein-only boosts, but could also shift subclass or isotype distributions in less favorable ways, such as reduced IgG3 or elevated IgA1. Finally, while boosting increases antibody magnitude, it does not automatically improve protective efficacy; careful consideration of immunogen design, delivery platform, boost schedule, and monitoring of subclass/isotype and functional quality is essential to optimize both the potency and longevity of vaccine-induced immunity (178, 232, 233).

ALVAC-based HIV vaccine trials, including RV144, and RV305, demonstrated strong safety and tolerability, with partial efficacy observed in RV144 (31% reduction in HIV acquisition) primarily linked to non-neutralizing antibodies and CD4^+^ T cell responses. Overall, ALVAC-HIV induces modest CD8+ T-cell responses in approximately 20–50% of recipients, so, strategies such as protein boosting, dendritic cell loading (218), and delayed boosting enhanced humoral and cellular immunity, including maturation of Env-specific memory B cells and CD8+ responses. These results highlight ALVAC’s potential as a safe, immunogenic platform capable of inducing immune correlates associated with reduced HIV risk, though further optimization of immunogen design and boosting strategies is needed for broader and more durable protection (178, 218, 232–236).

### New York vaccinia virus

#### New York vaccinia virus biology

NYVAC is a highly attenuated vaccinia virus vector derived from the Copenhagen strain by deleting 18 non-essential ORFs, including genes encoding thymidine kinase, ribonucleotide reductase, hemagglutinin, and several immune evasion proteins. Engineered to express heterologous antigens, it has shown safety and immunogenicity in clinical trials (237). The optimized variant, NYVAC-KC, restores replication competence in human cells through reintroduction of the K1L and C7L host range genes and enhances immune activation by deleting a viral type I IFN inhibitor. Compared to parental Copenhagen and NYCBH strains, NYVAC-KC exhibits higher transgene expression while maintaining attenuation, as verified by intracranial inoculation in newborn mice (238).

Although related, NYVAC and MVA differ in *in vitro* biology: NYVAC induces cytopathic effects in both permissive and non-permissive cells, whereas MVA produces minimal cytopathic effect (CPE) due to deletions affecting ~15% of its genome (239). NYVAC yields lower titers in BHK-21 cells and suppresses protein synthesis via increased eIF-2α phosphorylation (Ser51) (240). Viral morphogenesis also diverges—MVA arrests after immature virion formation, while NYVAC halts at or before this stage due to missing late proteins—and NYVAC triggers apoptosis in ~42% of infected cells within 24 h through a caspase-dependent mechanism (239).

### NYVAC-based HIV-1 vaccines

NYVAC-based HIV-1 vaccine candidates have been tested in a wide range of preclinical and clinical settings, consistently showing encouraging safety and immunogenicity outcomes (33, 241–246). In non-human primate studies, NYVAC vectors have demonstrated stronger immunogenicity than ALVAC, particularly in the induction of HIV-specific cellular immune responses. These findings highlight NYVAC’s potential as a vaccine platform based on its safety profile, capacity to elicit broad immune responses, and flexibility for genetic modifications to enhance efficacy (247, 248).

The NYVAC-C (vP2010) (244) vaccine expressing HIV subtype C gag, pol, env and nef antigens developed by Sanofi Pasteur was the first NYVAC construct tested in humans (166). In the EV01 phase I trial conducted in Lausanne and London, HIV-negative adults at low infection risk received two intramuscular doses of NYVAC-C or placebo. The vaccine was well tolerated, with no serious adverse events or discontinuations. Among ten participants evaluated for T-cell responses, 50% showed positive IFN-γ ELISpot activity, while humoral responses to gp140 CN54 were modest. In addition, the vaccine induced both CD4^+^ and CD8^+^ T-cell responses, which were polyfunctional, evidenced by T cells secreting both IFN-γ and IL-2.

Another Phase I clinical trial (EV02) (246) evaluated the safety and immunogenicity of a DNA-C prime followed by a NYVAC-C boost compared to NYVAC-C alone. The study demonstrated that both regimens were well tolerated and that priming with DNA enhanced HIV-1–specific T- and B-cell immune responses. These findings suggest that the DNA-C prime–NYVAC-C boost regimen elicits T-cell responses comparable to those induced by the trivalent Merck Ad5 gag-pol-nef vaccine. Notably, the DNA/NYVAC regimen induces predominantly Env-specific responses, which may offer an advantage in light of recent Phase IIb results from the MRK-Ad5 trial.

The HVTN 078 phase Ib trial, for the first time (249), assessed heterologous prime–boost combinations of NYVAC-B and recombinant Ad5 vectors. Ad5-seronegative, HIV-negative adults were randomized into regimens varying the order and dose of NYVAC-B and rAd5 administration. NYVAC-B expressed gp120 from HIV-1 BX08 and *gag-pol-nef* from HIV-1 IIIB. Overall, the vaccines were safe and well tolerated, eliciting strong HIV-specific immune responses, with 100% of participants showing binding and neutralizing antibody responses and over 85% responding in both T-cell subsets. Notably, CD4^+^ T-cell responses predominated in this trial, which is unusual for regimens including rAd5. CD8^+^ T-cell responses were slightly lower in HVTN 078 compared with HVTN 054, where a single dose of the same rAd5 was administered (250).

The HVTN 096 trial (251) further assessed DNA- and NYVAC-based regimens in healthy adults in Lausanne in a phase Ib, randomized, double-blind experiment. The study evaluated four experimental HIV vaccine regimens, each incorporating a booster with the NYVAC (NYVAC-HIV-PT1 (expressing HIV-1 clade C 96ZM651gp140) and NYVAC-HIV-PT4 (expressing HIV-1 clade C 96ZM651Gag fused to HIV-1 clade C CN54PolNef)) and DNA (DNA-HIV-PT123; expressing HIV-1 clade C 96ZM651gp140, 96ZM651Gag, and CN54PolNef) combination. HVTN 096 was designed to determine whether co-administering gp120 Env protein at priming could accelerate and enhance the generation of antibody responses associated with protection in RV144. The co-administration strategy resulted in rapid and strong induction of these antibodies, with V1/V2-directed IgG responses comparable to RV144 and binding IgG responses to gp120/gp140 similar to or exceeding those observed in HVTN 100 and RV144. Overall, all four vaccine regimens in HVTN 096 were immunogenic, with T-cell responses and some antibody responses higher in regimens that included the DNA vector. Importantly, co-administration of Env protein at priming not only accelerated the development of protective antibody responses but also improved their durability, maintaining broader antibody coverage over 18 months compared with regimens without protein co-administration (252).

Overall, these trials show that NYVAC vectors are safe, well tolerated, and capable of inducing potent immune responses. In the EV01 phase I trial, two doses of NYVAC-C expressing HIV subtype C gag, pol, env, and nef elicited both CD4^+^ and CD8^+^ T-cell responses, which were polyfunctional as indicated by simultaneous IFN-γ and IL-2 secretion, although humoral responses to gp140 were modest. The EV02 trial further demonstrated that priming with DNA followed by a NYVAC-C boost enhanced both T- and B-cell responses compared with NYVAC-C alone, with Env-dominant responses comparable to other vector-based regimens, highlighting the utility of NYVAC in eliciting cellular immunity. In HVTN 078, NYVAC-B in combination with rAd5 was well tolerated and induced strong HIV-specific immune responses. Across different heterologous prime–boost regimens, NYVAC-B contributed to high binding and neutralizing antibody titers and elicited T-cell responses in over 85% of participants, with CD4^+^ T-cell responses predominating. In HVTN 096, NYVAC-based boosts (NYVAC-HIV-PT1 and NYVAC-HIV-PT4) elicited rapid and strong antibody responses when combined with DNA primes and co-administered gp120 Env protein, demonstrating that NYVAC vectors effectively contribute to both T-cell and antibody immunity and can enhance the speed, magnitude, and durability of vaccine-induced responses.

To summarize, Pox-based HIV vaccines have a strong safety record in humans and elicit potent cellular and humoral responses across multiple phase I/II studies. Trials using MVA- and NYVAC-vectored constructs repeatedly show good tolerability and induction of CD4^+^/CD8^+^ T cells and binding antibodies; therapeutic and prophylactic MVA regimens generated durable T-cell memory in many volunteers, and NYVAC formulations produced measurable cellular and humoral immunity in phase I trials. These platform-level results have established pox vectors as reliable, well-tolerated carriers for HIV antigens. However, efficacy readouts have been modest. The ALVAC (canarypox) prime + AIDSVAX boost regimen achieved the only reproducible signal of protection to date — ~31% reduction in HIV acquisition in RV144 — but did not reduce viral load in breakthrough infections and the effect waned over time, highlighting limitations in potency, breadth and durability. Other pox-vector trials (including various NYVAC/MVA regimens and different prime-boost schedules) have improved immunogenicity but have not yet translated into clear, reproducible protection, prompting work on optimized priming (e.g., DNA primes), altered inserts (mosaic/consensus immunogens), and combination strategies to enhance breadth and mucosal immunity. In short: pox vectors are safe and immunogenic but, by themselves, have provided only modest clinical efficacy—future success will depend on superior antigen design and smarter prime-boost/delivery regimens.

## Concluding remarks

Despite decades of intensive effort, immune correlations of protection against HIV-1 remain elusive, underscoring the need for more refined immunological assays and analytical frameworks capable of capturing protective immunity in all its complexity. Nevertheless, accumulated evidence now converges on several essential principles: a successful HIV-1 vaccine—whether prophylactic or therapeutic—must induce durable, multifaceted immunity that can constrain viral replication upon exposure, contend with the extraordinary global diversity of circulating strains, and establish effective immune surveillance at mucosal portals of entry. Such protection will likely require the coordinated engagement of humoral and cytotoxic T-lymphocyte responses, early containment of reservoir seeding, and strong immunity within genital and rectal tissues where transmission is initiated.

Within this landscape, viral vector platforms have played a defining role in shaping contemporary HIV vaccine science. Replication-incompetent vectors, favored for their safety, have demonstrated that transient antigen expression can nonetheless drive sustained adaptive immunity, particularly when leveraged through carefully designed prime–boost regimens. Conversely, replicating vectors offer conceptual advantages through prolonged antigen availability and heightened immunogenicity, albeit with additional considerations regarding immune activation and safety. The central challenge moving forward is therefore not only to define the immune responses that confer protection, but to precisely engineer immunogens and delivery systems that elicit these responses systemically and at mucosal sites without inadvertently increasing susceptibility to infection.

Clinical evaluation of adenoviral and poxviral vectors over the past two decades has generated an unparalleled body of evidence informing these efforts. Although landmark trials such as STEP, Phambili, and HVTN 702 did not demonstrate durable protection against acquisition, they provided critical insights into vector-specific immunogenicity, the impact of pre-existing immunity, and the qualitative features of vaccine-induced T-cell responses. Adenoviral vectors—including Ad5, Ad26, and chimpanzee-derived constructs—have consistently shown strong cellular immunogenicity, particularly against conserved HIV antigens, while poxvirus vectors such as ALVAC and MVA have combined excellent safety with modest efficacy, most notably in RV144, reinforcing the value of heterologous prime–boost strategies.

While heterologous regimens have yet to achieve sustained clinical efficacy, they remain central to HIV vaccine development, reliably expanding the breadth and magnitude of immune responses and serving as a platform for iterative innovation. Collectively, the lessons learned from adenoviral and poxviral vector trials have crystallized the guiding principles of modern vaccine design: minimizing seroprevalence constraints, optimizing immunogen composition, and deploying advanced assays to identify correlates of protection. These foundational insights have directly informed the emergence of next-generation modalities, including mRNA vaccines and engineered viral vectors, establishing benchmarks for safety, immunogenicity, and regimen architecture.

As the field advances toward increasingly sophisticated vaccine strategies, the legacy of adenovirus- and poxvirus-based HIV-1 vaccines remains integral. Far from representing endpoints, these platforms have served as essential proving grounds—defining the challenges, revealing the possibilities, and continuing to shape the path toward a durable and protective HIV-1 vaccine.
